# Clinical validation of a wireless patch-based polysomnography system

**DOI:** 10.5664/jcsm.11524

**Published:** 2025-05-01

**Authors:** Christian Viniol, Wolfgang Galetke, Holger Woehrle, Georg Nilius, Christoph Schöbel, Winfried Randerath, James Leiter, Sebastian Canisius, Hartmut Schneider

**Affiliations:** ^1^ Department of Pneumology, Universitätsklinikum Gießen und Marburg GmbH, Marburg, Germany; ^2^ VAMED Klinik Hagen-Ambrock, Hagen, Germany; ^3^ Lungenzentrum Ulm, Ulm, Germany; ^4^ Klinikum Dortmund gGmbH, Dortmund, Germany; ^5^ Department of Pneumology, Universitätsmedizin Essen Ruhrlandklinik, Essen, Germany; ^6^ Faculty of Sleep and Telemedicine, Universitätsmedizin Essen Ruhrlandklinik, Essen, Germany; ^7^ Krankenhaus Bethanien, Solingen, Germany; ^8^ Department of Molecular and Systems Biology, Geisel School of Medicine, Hanover, New Hampshire; ^9^ ACliRA Consulting ApS, Tønder, Denmark; ^10^ American Sleep Clinic, Frankfurt, Germany; ^11^ Onera Health, Eindhoven, Netherlands; ^12^ Johns Hopkins University, Baltimore, Maryland

**Keywords:** polysomnography, home PSG, respiratory events, sleep staging, sleep-disordered breathing

## Abstract

**Study Objectives:**

Onera Health has developed the first wireless, patch-based, type-II polysomnography (PSG) system, the Onera Sleep Test System, to allow studies to be performed unattended at the patient’s home or in any bed at a medical facility. The goal of this multicenter study was to validate data collected from the patch-based PSG to a traditional PSG for sleep staging and apnea-hypopnea index.

**Methods:**

Simultaneous traditional PSG and patch-based PSG study data were obtained in a sleep laboratory from 206 participants with a suspected sleep disorder recruited from 7 clinical sites. Blinded, randomized scoring of the traditional PSG and patch-based PSG recordings was completed according to *The AASM Manual for the Scoring of Sleep and Associated Events: Rules, Terminology and Technical Specifications* (Version 2.6) criteria by 3 independent scorers.

**Results:**

Concordance correlation coefficients were high between the patch-based device and traditional PSG across essential sleep and respiratory variables—total sleep time (.87); wake (.84); non-rapid eye movement (REM) (.80); non-REM sleep stage 1 (N1) (.72); non-REM sleep stage 2 (N2) (.71); non-REM sleep stage 3 (N3) (.64); REM (.80); and apnea-hypopnea index (AHI) (.94). There was substantial agreement between epoch sleep staging scored on the patch-based device and traditional PSG (average Cohen’s kappa of 0.62 ± 0.13 across all scorers).

**Conclusions:**

The patch-based type-II PSG had a similar performance on sleep staging and respiratory variables when compared to traditional PSG, thus making it possible to use the patch-based PSG for a routine PSG study. These results open the possibility of performing unattended PSG studies efficiently and accurately outside the sleep laboratory improving access to high quality sleep assessments for patients with sleep disorders.

**Clinical Trial Registration:**

Registry: ClinicalTrials.gov; Name: Validation Study of a Patch-based PSG System; URL: https://clinicaltrials.gov/study/NCT05310708; Identifier: NCT05310708.

**Citation:**

Viniol C, Galetke W, Woehrle H, et al. Clinical validation of a wireless patch-based polysomnography system. *J Clin Sleep Med*. 2025;21(5):813–823.

**BRIEF SUMMARY**

**Current Knowledge/Study Rationale:** Increasing demand for sleep studies (estimated 12% growth by 2032) will exceed available resources such as certified personnel, polysomnography (PSG) equipment and laboratory space. PSG devices that are simple, and can be used independently by patients at home, could address this issue.

**Study Impact:** The results of this study suggest that patch-based PSG is a viable, clinical alternative to a traditional PSG, which could enable patients to perform an unsupervised PSG study at home.

## INTRODUCTION

The demand for sleep diagnostic studies in developed countries is expected to grow by 12.3% between 2024 and 2032.^1^ This estimate includes studies being performed for all major sleep disorders: insomnia, restless legs syndrome, narcolepsy, rapid eye movement (REM) disorders, bruxism, and obstructive sleep apnea. The increase in demand for in-laboratory polysomnography (PSG) cannot be met with the resources currently available. In the United States, multidisciplinary sleep clinics are common, but the number of PSGs performed in these centers is capped by the availability of traditional PSG equipment and trained personnel (both sleep laboratory technicians and board-certified sleep physicians). Neither the technical nor personnel limitations are likely to be addressed at the rate required to meet the clinical demand for PSG.

The recent introduction of portable PSG devices has enabled acquisition of type-II PSG studies in the patient’s home (a type-II PSG is defined as an unattended PSG that uses the same monitoring sensors as a traditional, attended, type-I PSG). However, uncertainty remains regarding the technical feasibility of this approach and whether patients prefer this to a traditional in-laboratory PSG. Furthermore, nominally portable PSG devices are not yet simple enough for a patient to perform a study unattended at home, as most currently deployed, common type-II PSG models require a technician’s intervention for device setup and device collection after the study—thus creating bottlenecks in the diagnostic process. Economic barriers to type-II PSG testing also exist, as reimbursement in the United States does not yet adequately cover type-II studies.

Onera Health has developed the first wireless, patch-based, type-II PSG system, the Onera Sleep Test System (Onera STS). This patch-based system was designed to overcome the technical and staffing limitations associated with a traditional, attended PSG by simplifying the device application process and using wireless protocols for sleep study data collection. The Onera STS consists of 4 sensors that adhere to specific body locations, which enable a 15-channel PSG study to be performed unattended by the patient at home or, due to its portable nature, in any bed at a medical facility.

There are notable differences in the design of the patch-based PSG system when compared to a traditional PSG. The primary difference is within the head sensor where electroencephalography (EEG) data are captured from forehead leads (frontal left and frontal right), which could theoretically affect scorers’ determination of sleep onset and their recognition of slow wave sleep. Additionally, upper airway electromyography (EMG) activity is assessed as muscle tone and activity of the masseter in the patch-based system rather than submental muscles as in the traditional PSG. Finally, the chest sensor utilizes bioimpedance technology in the patch-based system rather than effort belts or inductance plethysmography.

The goal of this multicenter study was to collect a robust data set to compare the performance of the patch-based PSG system to standard of care, in-laboratory PSGs in recording sleep and respiratory data scored according to the American Academy of Sleep Medicine (AASM) 2020 *The AASM Manual for the Scoring of Sleep and Associated Events: Rules, Terminology and Technical Specifications* (Version 2.6) rules.^2^ The hypothesis being tested was that scoring of sleep staging and respiratory events would not differ between the 2 measurement systems.

## METHODS

### Objectives and outcomes

The primary objective of the study was to compare sleep-stage and respiratory scoring between the patch-based PSG system and a traditional PSG system. For this analysis the following sleep metrics were compared: total sleep time (TST), wake time, time in non-REM (NREM) sleep stage 1 (N1), NREM sleep stage 2 (N2), NREM sleep stage 3 (N3), REM and NREM, sleep efficiency, sleep onset latency, wake after sleep onset, REM onset latency, and the apnea-hypopnea index (AHI). Agreement was assessed using concordance correlation coefficients (CCCs), Bland-Altman plots and Cohen’s kappa agreement.^3^

A secondary outcome of this study was to compare device setup time by the sleep technician.

### Study design

A multicenter study was conducted across 7 clinical sites in Germany. All participants who were referred for a sleep study were asked to participate in the clinical study. The participating sites were (1) VAMED Klinik Hagen-Ambrock, (2) Universitätsklinikum Gießen und Marburg, (3) Lungenzentrum Ulm, (4) Evang. Kliniken Essen-Mitte, (5) Universitätsmedizin Essen Ruhrlandklinik, (6) Krankenhaus Bethanien, Solingen, and (7) the American Sleep Clinic, Frankfurt. The study was approved under reference number 2021-2521-evBO by the ethics committee at the chamber of physicians Hessen and local ethics committees as required. Consecutive participants, who met the inclusion criteria and consented to participate, underwent a single night sleep evaluation in a sleep laboratory using both the patch-based PSG system and a traditional PSG system recorded simultaneously.

### Patients’ rights protection statement

All procedures followed were in accordance with the ethical standards of the committees responsible for human experimentation (institutional and national) and with the Helsinki Declaration of 1975 (in its most recently amended version). Informed consent was obtained from all patients included in the study.

### Inclusion

Clinical site personnel reviewed each individual’s medical history for eligibility. Participants were considered eligible if they met the inclusion criteria (18 years and older; either referral for a suspected sleep disorder requiring a sleep diagnostic study, or a planned follow-up PSG for an established sleep disorder [without therapy]), did not present any exclusion criteria (inability to provide informed consent; history of allergic reactions to adhesives or hydrogels or a family history of adhesive skin allergies; severe skin conditions at sites of patch administration; implanted cardiac stimulator or diaphragmatic pacer; an abnormality that made the individual ineligible for inclusion; exposure to high-frequency surgical equipment, near strong magnetic fields, external cardiac stimulators) and consented to participate.

### Devices

#### Onera STS

**Figure 1:**
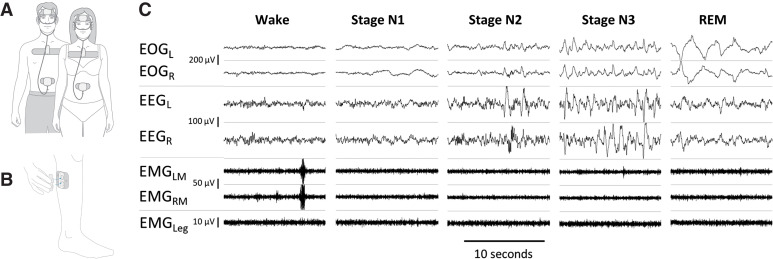
The patch-based type-II PSG system and example signal output. The patch-based type-II PSG system consists of 4 sensors located on the head, chest, abdomen **(A)** and leg **(B)**. The patch-based sensors consist of disposable patches and reusable pods that snap into the patches for data collection **(B)**. Example signal output of data collected using the patch-based type-II PSG system during different sleep stages can be seen in **(C)**. EOG_L_ = electrooculography left, EOG_R_ = electrooculography right, EEG_L_ = electroencephalography frontal left, EEG_R_ = electroencephalography frontal right, EMG_LM_ = electromyography of the masseter left, EMG_RM_ = electromyography of the masseter right, EMG_Leg_ = electromyography at the antierior tibialis, PSG = polysomnography, REM = rapid eye movement.

The Onera STS (legal manufacturer Onera B.V., Eindhoven, Netherlands) is a wearable patch-based type-II PSG system for measuring physiological signals during a sleep study conducted at home.^4^ The patch-based PSG system consists of 4 disposable patches applied to the forehead, upper chest, abdomen, and lower leg, and reusable pods that click into the patches to measure the physiological signals required for a sleep study, see **Figure 1A** and **Figure 1B**. The head sensor (patch and pod system combined) measures electrooculography (EOG) (left and right), EEG (frontal left and frontal right), EMG masseter (left and right), and oxygen saturation (reflective pulse oximeter on the forehead). The chest sensor measures electrocardiography, respiratory flow (bio-impedance based measurement), respiratory effort (bio-impedance based measurement), activity (accelerometer-based measurement from the chest), snoring level (via a microphone on chest), and body position (accelerometer-based measurement from the chest). The flow sensor measures respiratory flow (nasal cannula). The leg sensor measures EMG from the anterior tibialis muscle. Examples of the signals from the patch-based PSG during different sleep stages can be found in **Figure 1C**. An Onera STS sleep study is uploaded to a secure Onera cloud-based data storage system that generates an Electronic Data Format (EDF) file of the data. Onera sleep study EDFs can be read using any sleep scoring software with some adjustments to display settings.

#### Traditional PSG

Traditional PSG studies were performed with the device routinely used at each participating sleep clinic (see **Table S1** in the supplemental material). Setup was standardized to include EEG (C3, C4, F3, F4, O1, and O2), left and right EOG (E1–M2 and E2–M1), chin EMG (anterior, left, and right positioning), airflow (nasal pressure), oxygen saturation, electrocardiography, breathing efforts (abdomen and thorax), sleeping position, audio and video recording of the entire night and optional tibialis EMG on both legs. Minor alterations of some of the electrode positions were made to accommodate the Onera STS head sensor, as previously described. These alterations did not impact the quality of the traditional PSG signal.

### Sleep study setup procedure

The technicians applied the Onera patches in the following order: head, chest, flow, leg, followed by the corresponding pod. The technician then applied the traditional PSG system, adjusting the position of the reference EEG and left and right EOG, to avoid interference with the Onera STS head sensor.

At bedtime, both devices were set to record, and the individual was allowed to go to sleep. The traditional PSG was monitored throughout the night as normal. Adjustments were made to the PSG by the technician if there was a lead-off or poor signal quality (as per the standard of care). It was not possible for the technicians to monitor the patch-based PSG throughout the night, so adjustments were not made to this system.

### Study acceptance criteria

If both the patch-based PSG and traditional PSG sleep study met the study acceptance criteria (see below), the recordings were anonymized, randomized and sent to 3 independent, certified sleep professionals, who used RemLogic software (version 1.3, Embla Systems Ltd., Kanata, Canada) to score the studies according to AASM guidelines (Version 2.6), 2020^2^: (1) the Johns Hopkins Center for Interdisciplinary Sleep Research and Education in Baltimore, MD, (2) Sleep Scoring Service LCC in Utah, and (3) a Registered Polysomnographic Technologist, independent sleep scorer in Switzerland. Sleep professionals were blinded to the patient identification and sleep study device used for each recording.

Following completion of a sleep study, the data were inspected by a licensed, independent sleep physician to assess signal quality and completeness. A successful study was defined as a study in which:

1. the patch-based PSG study AND the traditional PSG study had ≥ 6 hours of time in bed AND
2. ≥ 4 hours of the study recording consisted of sleep (TST) AND
3. all signals essential for scoring according to AASM criteria were present AND
4. all signals essential for scoring according to AASM criteria were of scorable quality for at least half of time in bed.

The essential signals for the sleep study following 2020 AASM guidelines were the oxygen saturation signal, breathing efforts (thorax and/or abdomen signal), airflow (nasal pressure, optional also thermistor in case of signal deficiencies in nasal pressure), 1 EEG channel (patch-based PSG) or 2 EEG channels (traditional PSG), 1 EOG channel (patch-based PSG) or 2 EOG channels (traditional PSG), EMG chin/masseter, and electrocardiography.^2^ Only the subset of signal variables necessary to assess the quantity and quality of sleep, are described here. Further manuscripts will be published describing other aspects of the signal set.

### Analysis of sleep summary statistics

The following variables were calculated: TST, wake time, time in N1, N2, N3, REM and NREM sleep, sleep efficiency, sleep onset latency, wake after sleep onset, REM onset latency, and AHI.

The test-retest performance of in-laboratory PSG indicates substantial variability.^5^^–^^7^ Therefore, to compare 2 diagnostic devices, a concordance correlation-based reduced major axis (RMA) regression seemed appropriate to recognize the simultaneous variation in x (gold standard) and y (test device). The CCC was calculated for all sleep variables to estimate agreement and precision in performance between the 2 devices. The CCC derived from RMA regression provides a measure of precision, and the bias correction factor provides a measure of how far the line of best fit differs from the line of identity. The CCC also provides measures of location shift in the intercept and scale shift—the amount of deviation of the calculated slope of the relationship between x and y from the line of identity.^8^ The Altman guidelines for CCC interpretation will be used for this analysis.^9^

We used the intraclass correlation coefficient (ICC) to assess reliability between devices and scorers. We used a 2-way mixed effects model in 2 separate analyses: (1) sleep scorers were treated as a fixed factor, and (2) sleep devices were considered a fixed factor.^10^

Bland-Altman plots were generated for each variable, which show the average deviation of the patch-based measurements from the traditional PSG as a function of the average of each pair of measurements as well as the 95% confidence interval of the differences between each pair of measurements. Both the CCC and Bland-Altman plots are shown for all sleep stages either in the body of the paper or in the supplement, **Figures S1–S8** in the supplemental material.

### Analysis of epoch-by-epoch agreement

We assessed the average agreement between the patch-based PSG and traditional PSG systems on a 30 seconds epoch-by-epoch basis using Cohen’s kappa.^11^ In addition, we calculated the average, pairwise interscorer kappa agreement for each device. A majority agreement was also calculated in which the consensus sleep stage among all 3 scores was determined for each sample in both the patch-based PSG and traditional PSG and compared across devices. Samples that had no consensus agreement were dropped.

All statistical analyses were conducted using R in RStudio (2023.12.1 Build 402, RStudio, PBC, Boston, MA) or using Python. No imputation was made for missing values.

### Sample size calculation

We used GLIMMPSE web-based software to estimate the study size (https://samplesizeshop.org/glimmpse-power-software/).^12^ The software is specifically designed for power analyses of hierarchical mixed effects models. Scorers were treated as a between-participants factor, and sleep device was considered a repeated, within-participants factor. Before the study began, we made conservative assumptions about effect sizes and error variances, and we assumed that measurements made on the traditional PSG and the patch-based PSG within a single individual were likely to be more highly correlated than measurements between scorers made on either of the devices. Power curves were generated for a range of systematic differences between devices of ± 5% of total events/h; the standard deviation of the normal distribution of AHI was 20 events/h, the standard deviation of the AHI determined by each reader was 5 events/h, and alpha = 0.5. The power curves indicated that 200–300 sleep studies would need to be compared to detect a 5% difference between AHI scores with a power > 0.8. The assumptions were excessively pessimistic largely because the standard deviation about the AHI of the study population was smaller than the value used in the simulations, and the average difference between studies was less than 5%.

## RESULTS

### Consort diagram

**Figure 2:**
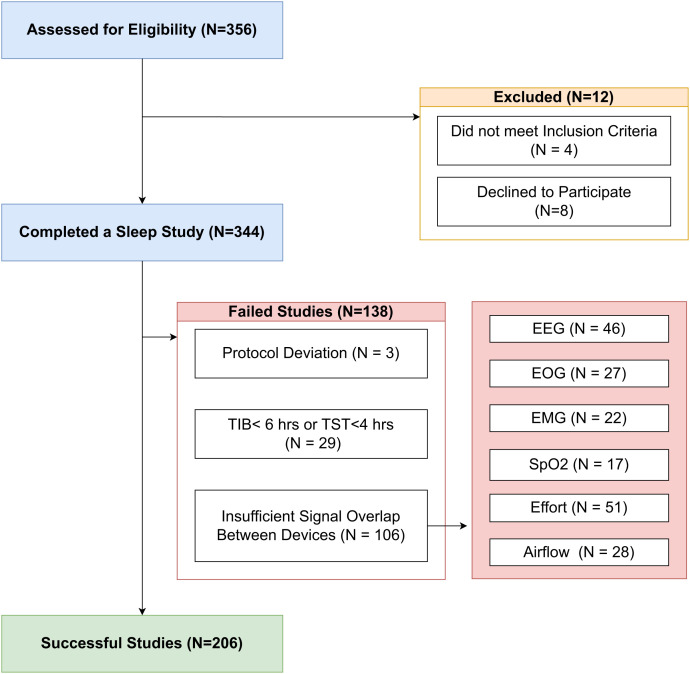
Consort diagram of participants included in the clinical study. A total of 206 participants met the criteria to be included in the study. The others were excluded due to consent, inclusion criteria, protocol deviations, sleep time requirements or insufficient signal overlap between devices. EEG = electroencephalography, EMG = electromyography, EOG = electrooculography, SpO_2_ = oxygen saturation, TIB = time in bed, TST = total sleep time.

Of the 356 participants recruited to participate in the study, a total of 344 participants consented and met the inclusion criteria to perform an overnight sleep study with the patch-based PSG and traditional PSG. A total of 206 participants met the acceptance criteria and were included in this analysis, see **Figure 2**.

### Demographics

The demographics of the 206 included participants and the 138 excluded participants can be found in **Table 1**. Enrolled participants represented a typical sleep laboratory population. Included and excluded participant demographics were not statistically significant. For information on site recruitment rates, see **Table S1**.

**Table 1:** Participant demographics.

Demographic/Clinical Characteristics	Included Patients	Excluded Patients
Age, years	50.9 ± 12.3	50.2 ± 13.3
≥ 65	21 (10.2%)	16 (11.6%)
Sex (male/female)	136/70 (66.0/34.0%)	92/46 (66.7/33.3%)
BMI (kg/m^2^)	30.2 ± 6.2	31.3 ± 6.2
Race		
Black or African American	6 (2.9%)	3 (2.2%)
Asian	0 (0.0%)	1 (0.7%)
White	133 (64.6%)	84 (60.9%)
Not reported/unknown	66 (32.0%)	48 (34.8%)
Other	1 (0.5%)	2 (1.4%)
Ethnicity		
African	6 (2.9%)	3 (2.2%)
European	135 (65.5%)	84 (60.9%)
Hispanic or Latino	0 (0.0%)	1 (0.7%)
Pacific Islander	0 (0.0%)	2 (1.4%)
Not reported/unknown	64 (31.1%)	48 (34.8%)
Other	1 (0.5%)	0 (0.0%)
Comorbidity		
Coronary artery disease	11 (5.3%)	8 (5.8%)
Depression	18 (8.7%)	7 (5.1%)
Diabetes	18 (8.7%)	9 (6.5%)
Heart failure	2 (1.0%)	0 (0.0%)
High cholesterol	18 (8.7%)	7 (5.1%)
Hypertension	74 (35.9%)	57 (41.3%)
Irregular heart rhythm	2 (1.0%)	0 (0.0%)
Obstructive lung disease	26 (12.6%)	20 (14.5%)
Seizures/epilepsy	3 (1.5%)	1 (0.7%)
Severe pain (on opioids)	3 (1.5%)	7 (5.1%)
Stroke	2 (1.0%)	1 (0.7%)
Tachy-brady syndrome	2 (1.0%)	2 (1.4%)
Thyroid diseases	34 (16.5%)	21 (15.2%)
Medication		
Alpha-1 adrenergic antagonist	4 (1.9%)	5 (3.6%)
Angiotensin-converting enzyme inhibitor	29 (14.0%)	26 (18.8%)
Antidepressant	20 (9.7%)	21 (15.2%)
Antidiabetic	16 (7.8%)	12 (8.7%)
Beta blocker	19 (9.2%)	15 (10.9%)
Benzodiazepine	1 (0.5%)	2 (1.4%)
Calcium channel blocker	18 (8.7%)	19 (13.8%)
Diuretic	13 (6.3%)	12 (8.7%)
Levothyroxine	30 (14.5%)	20 (14.4%)
Opiates	3 (1.5%)	7 (5.1%)

The included participant demographics (n = 206) and excluded participant demographics (n = 138) are reported in 2 manners as (1) count (percent of total individuals), n (%), or as (2) the mean and standard deviation of all individuals. There were no statistically significant differences between the included and excluded patient population evaluated using the appropriate test (Chi-squared test, Fisher’s exact test or unpaired *t* test). BMI = body mass index.

### Primary endpoint

#### Sleep summary statistics

The TST, NREM sleep, REM sleep, and AHI CCC and Bland-Altman plots are found in **Figure 3** and **Figure 4**. CCC and Bland-Altman plots for wake, N1, N2, N3, wake after sleep onset, REM sleep latency, sleep onset latency, and sleep efficiency can be found in **Figures S1–S8**.

**Figure 3:**
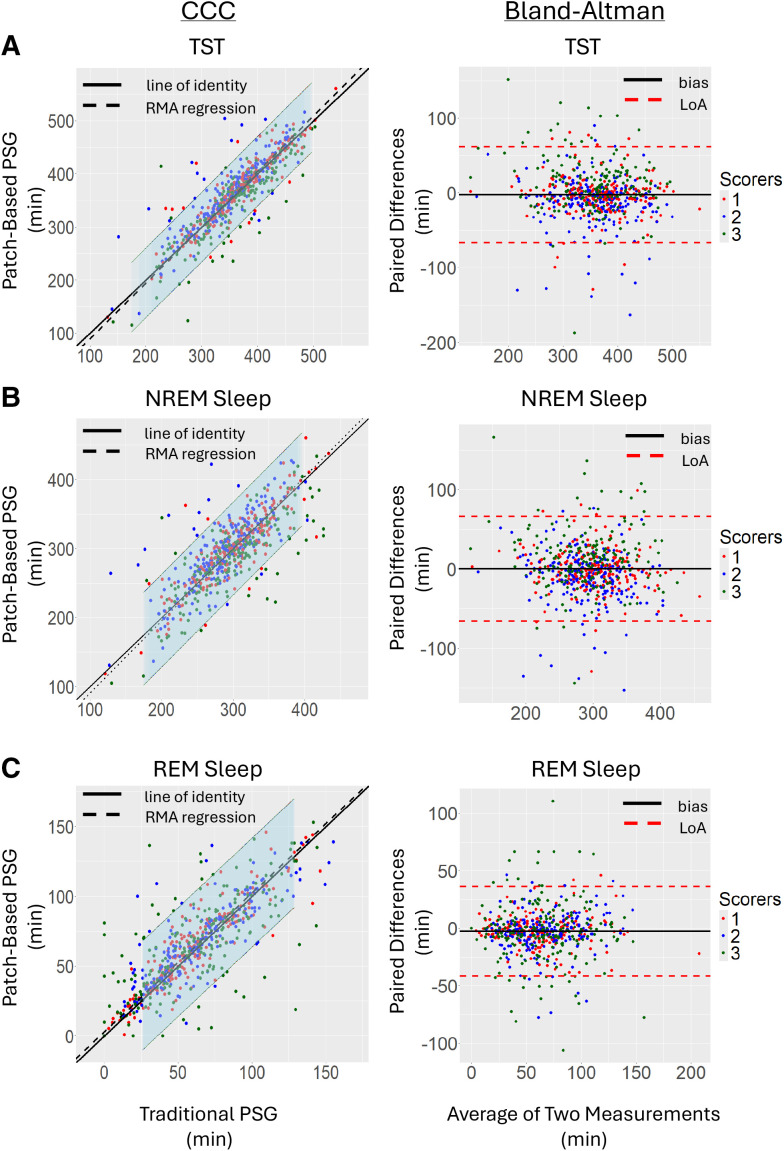
CCC and Bland-Altman plot of sleep summary variables. Left: CCC for TST **(A)**, NREM **(B)**, and REM **(C)** from 3 scorers comparing the traditional PSG to the patch-based PSG. Each scorer is represented by a different colored dot. The line of identity is shown as a solid black line, and the RMA regression is shown as a dotted line. Note that the RMA regression and the line of identity are not detectably different for all sleep variables (the 95% confidence interval of the RMA regression is shown in light blue). Right: Bland-Altman plots for the 3 sleep variables. The average offset (bias) between measurements derived from the 2 devices is shown as a solid black line. There is virtually no bias for all sleep variables. The LoA (the 95% confidence interval of the differences between the 2 measurement methods) are shown as dashed, red lines. CCC = concordance correlation coefficient, LoA = limits of agreement, NREM = non-rapid eye movement, PSG = polysomnography, REM = rapid eye movement, RMA = reduced major axis, TST = total sleep time.

**Figure 4:**
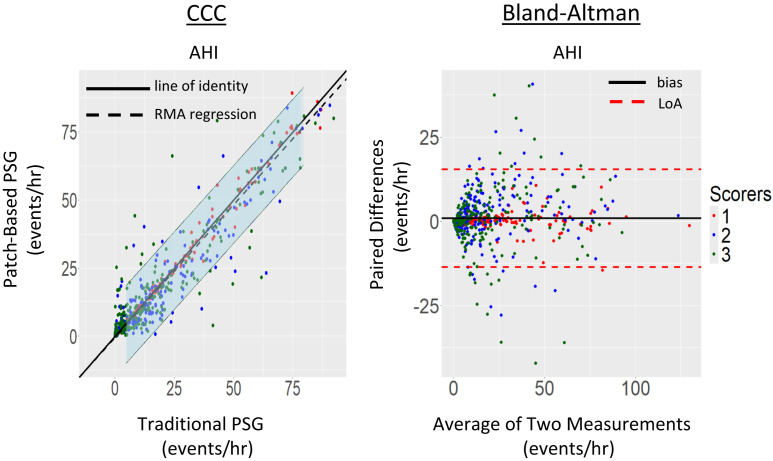
CCC and Bland-Altman plot of AHI. Left: CCC analysis of AHI (events/h) using the 4% rule from 3 scoring centers. Right: Bland-Altman plot of AHI. Figure conventions as described in **Figure 3**. AHI = apnea-hypopnea index, CCC = concordance correlation coefficient, LoA = limits of agreement, PSG = polysomnography, RMA = reduced major axis.

##### Interdevice agreement:

A summary of the CCC analysis results can be found in **Table 2**. Overall, we see high correlation in all sleep variables (> .70) except for N3 (.64). The highest CCC was found to be in TST (.87), wake (.84), sleep efficiency (.83) and wake after sleep onset (.82), indicating that the patch-based PSG performs well in comparison to a traditional PSG system in detection of wake and sleep. The worst performing variables were N3 (.64), N2 (.71), and N1 (.72). This is consistent findings in the literature where there is lower agreement between NREM sleep stages for sleep scorers and across devices.^13^^,^^14^ However, when comparing total NREM time across devices, the correlation was higher (.80), indicating that although there is lower agreement in individual stages of the NREM, overall, the agreement that the individual is in NREM sleep is high. The performance of REM time was also high in comparison to the traditional PSG (.80). The correlation was highest for the AHI (.94).

**Table 2:** CCC of sleep variables.

Sleep Event	CCC	Scale Shift	Location Shift	Bias Correction Factor
TST	.87	1.05	0.04	1.00
Wake	.84	1.02	−0.07	1.00
N1	.72	0.84	−0.20	0.97
N2	.71	1.03	0.24	0.97
N3	.64	1.01	−0.20	0.98
NREM	.80	1.05	0.00	1.00
REM	.80	0.99	0.09	1.00
Sleep efficiency	.83	1.04	0.04	1.00
WASO	.82	1.01	−0.12	0.99
REM sleep latency	.70	1.02	−0.02	1.00
Sleep onset	.80	1.03	0.07	1.00
AHI	.94	0.97	−0.04	1.00

Results from the CCC analysis for all sleep variables in this analysis comparing the traditional PSG to the patch-based PSG. The CCC represents the correlation between devices as calculated by RMA regression (utilizing the variation in both the x- and y-variables). The CCC derived from RMA regression provides a measure of precision, and the bias correction factor provides a measure of how far the line of best fit differs from the line of identity. The CCC also provides measures of location shift in the intercept and scale shift—the amount of deviation of the calculated slope of the relationship between x and y from the line of identity. AHI = apnea-hypopnea index, CCC = concordance correlation coefficient, N1 = NREM sleep stage 1, N2 = NREM sleep stage 2, N3 = NREM sleep stage 3, NREM = non-rapid eye movement, REM = rapid eye movement, TST = total sleep time, WASO = wake after sleep onset.

The scale shift, location shift and bias correction all indicate that the regression line between the patch-based outcomes and traditional PSG outcomes was close to the line of identity. The scale shift and the bias correct are close to 1, and the location shift is close to 0.

The CCC provides more detail than Pearson’s correlation coefficient and was nearly identical (difference < .03) for all analyzed sleep variables.

##### TST:

In **Figure 3A**, the CCC is high, and the regression of the patch-based PSG on the traditional PSG is close to the line of identity; the slope is not different from 1 (95% confidence interval of the slope of the regression, shown in light blue encompasses the line of identity), and the bias is small. Additionally, there is a random distribution of data from each scoring center. There is a slight shift in the RMA from the line of perfect concordance, indicating that there is a slight underestimation by the patch-based PSG of TST in lower TSTs and a negligible overestimation of TST in the patch-based PSG at higher TSTs. When looking at the Bland-Altman plot, the line of bias is close to 0 (there is no consistent variation in the estimation of TST across the sleep studies and scorers), and the distribution of differences is homogeneous across the average values (the colored dots are evenly distributed and interspersed across the range of average scores with no change in bias as a function of the magnitude of the average; the slope of the bias is close to 0).

##### NREM:

In **Figure 3B** there is a random distribution of data from the scoring. There is a slight bias in the RMA line of regression from the line of identity, indicating that there is a small overestimation of NREM time at higher average NREM values by the patch-based PSG in comparison to the traditional PSG (the same outcome is seen in the Bland-Altman plot where the bias is slightly less than 0). However, this slight bias is not significant in that the 95% confidence interval for the RMA regression of the patch-based NREM sleep time and the traditional PSG NREM sleep time includes the line of identity. When looking at the Bland-Altman plot, we see that the line of bias is slightly less than 0, although the average difference between devices is clinically negligible. The variability in the Bland-Altman plot is consistent across the NREM time range and has a random distribution among scoring centers as well.

##### REM:

In **Figure 3C** there is a random distribution of data from the scoring centers (no consistent bias from 1 site). There is a slight shift in the RMA from the line of perfect concordance, indicating that there is a small overestimation by the patch-based PSG of REM sleep time in comparison to the traditional PSG. Nevertheless, the line of identity is well within the 95% confidence interval for the RMA regression of the patch-based REM sleep time and the traditional PSG REM sleep time. When looking at the Bland-Altman plot, we see that the line of bias is close to 0, indicating that the average difference between devices is negligible. The variability in the Bland-Altman plot is consistent across the REM Time range and has a random distribution among scoring centers as well. Both plots indicate good agreement among scorers when comparing estimates of REM time derived from the 2 recording methods.

##### AHI:

In addition to sleep staging metrics, the AHI, using the 4% rule,^2^ was also investigated, see **Figure 4**. The CCC analysis shows near perfect agreement with the line of identity. The scorers provided remarkably similar estimates of the AHI across a range of AHI values regardless of the source of the recording that they were evaluating. When looking at the Bland-Altman plot, we see that the line of bias is close to 0, indicating that the average difference between devices is negligible and clinically insignificant. The variability in the Bland-Altman plot is consistent across the range of AHI values and has a random distribution among scoring centers as well. Among all the variables examined, the agreement among scorers was greatest for the AHI.

##### Interscorer reliability:

**Table 3** investigates ICC values in 2 manners: (1) sleep scorers were treated as a fixed factor (labeled as pooled data in **Table 3**), and (2) sleep devices were considered a fixed factor (traditional PSG and patch-based PSG).^10^ A high correlation in the pooled data indicates that there were no differences between the scoring of the patch-based PSG and traditional PSG device across scorers—indicating that scorers tend to have similar scoring patterns on both the traditional PSG and patch-based PSG. This correlation accounts for interscorer reliability as well as variability as a result of the self-reported nature of scoring sleep and respiratory events in PSG records. The results of this analysis showed all sleep variables to have a high ICC (> .70).

**Table 3:** ICC of sleep variables.

Sleep Event	Pooled Data	Traditional PSG	Patch-Based PSG
TST	.93	.96	.93
Wake	.92	.95	.90
N1	.85	.78	.72
N2	.84	.79	.74
N3	.79	.79	.72
NREM	.89	.92	.89
REM	.89	.92	.89
Sleep efficiency	.91	.95	.90
WASO	.91	.93	.86
REM sleep latency	.82	.85	.84
Sleep onset	.89	.90	.89
AHI	.97	.98	.97

Pooled data represents the ICC analysis performed using sleep scorers as a fixed factor. In the other 2 analyses sleep device type was used as a fixed factor. AHI = apnea-hypopnea index, CCC = concordance correlation coefficient, ICC = intraclass correlation coefficient, N1 = NREM sleep stage 1, N2 = NREM sleep stage 2, N3 = NREM sleep stage 3, NREM = non-rapid eye movement, REM = rapid eye movement, TST = total sleep time, WASO = wake after sleep onset.

##### Interscorer variability:

For the second ICC analysis, the ICC was calculated within each device type (**Table 3**, traditional PSG and patch-based PSG). A high correlation indicates that there was good agreement among the 3 scorers on the sleep records derived from the same device. We see that there were high levels of agreement (> .70) for all variables for both the traditional PSG and patch-based PSG devices. Overall, the lowest and highest interscorer agreements were consistent across both devices indicating that sleep variables were equally easily identified from both devices.

#### Epoch agreement

##### Epoch sleep stage interdevice agreement:

We assessed the agreement between the patch-based PSG and traditional PSG systems on an epoch-by-epoch basis, see **Table 4**, A. Using Cohen’s original interpretation of the Cohen’s kappa scores,^11^ Scorer 1 (0.59) had a moderate agreement between the patch-based PSG and traditional PSG and Scorers 2 and 3 (0.64, 0.64) had substantial agreement between the patch-based PSG and traditional PSG. The overall scorers statistic is the average of all Cohen’s kappas across all studies and all scorers when comparing the traditional PSG and patch-based PSG and showed substantial agreement (0.62). The majority agreement kappa showed the highest agreement 0.75 (substantial) which was an unsurprising finding as it reduces interscorer variability due to scoring style and error. Less than 5% of samples were dropped due to a lack of consensus from all 3 scorers.

**Table 4:** Device agreement and interscorer variability using Cohen’s kappa and accuracy.

A.	Scorer 1	Scorer 2	Scorer 3	Overall Scorers	Majority Agreement
Cohen’s kappa	0.59 ± 0.13	0.64 ± 0.13	0.64 ± 0.15	0.62 ± 0.13	0.75 ± 0.10
Accuracy (%)	71.39 ± 9.27	75.05 ± 9.12	75.94 ± 9.98	74.12 ± 9.56	83.74 ± 6.45
**B.**	**Scorer 1–2**	**Scorer 1–3**	**Scorer 2–3**		
Patch-based PSG	0.58 ± 0.13	0.57 ± 0.13	0.62 ± 0.13		
Traditional PSG	0.63 ± 0.14	0.63 ± 0.14	0.64 ± 0.13		

**(A)** Cohen’s kappa and accuracy of epoch-by-epoch agreement between the traditional PSG and patch-based PSG. Agreement between devices is reported for each of the 3 scorers as well as the overall average agreement across all scorers. The majority agreement is defined as the agreement between devices using a majority vote of the 3 scorers for each sample’s sleep stage.** (B)** Interscorer variability reported using Cohen’s kappa for pairwise agreement on each device per epoch. PSG = polysomnography.

##### Epoch sleep stage interscorer agreement:

To put the Cohen’s kappa agreement of the traditional PSG and the patch-based PSG into context, we must also look at the epoch interscorer agreement on each device, as shown in **Table 4**, B. Overall, we see comparable results to **Table 4**, A, indicating that the difference in performance across devices is not different from the difference between scorers on the same device. **Table 4**, B shows moderate to substantial agreement between scorers.

### Secondary endpoints

#### Application time

The average application time of the traditional PSG was 19.5 ± 6.8 minutes. The average application time of the patch-based was reported as 5.8 ± 1.8 minutes. The average application time of the patch-based PSG was less than one-third of the average time it took to apply the traditional PSG system.

### Protocol deviations

There were 3 protocol deviations in the study. In 1 case the patient was administered CPAP while using the patch-based PSG device and therefore the study was excluded from the analysis. In the other 2 cases the devices were not started simultaneously at the lights-off time resulting in a protocol deviation. The studies were excluded from the analysis.

#### Adverse events

Patients with known device contraindications were excluded from the study. A total of 23 adverse events were reported in the 356 patients included in the safety cohort. Twenty-two patients had 1 adverse event each, and 1 patient experienced 2 adverse events, making for an event rate of 6.2%. None of the reported events were categorized as serious. Of the 23 reported adverse events, 20 were caused by the patch-based PSG and 1 was possibly caused by the traditional PSG; therefore, the rate of events possibly related to the patch-based PSG was 5.9%. The reported events with a causal relationship to the device all fell within the same category: skin reddening, skin irritation and allergic reaction. In all but 1 instance, these reactions were short-lived. In the remaining case, the patient experienced a prolonged skin allergic reaction lasting more than 1 week. All events with a causal relationship to the device fell within the expected categories of adverse events and incident rate listed by the manufacturer. The 2 events with no causal relationship to the patch-based PSG consisted of 1 COVID-19 infection and 1 infection with the common cold. All adverse events were evaluated until a resolution was noted or the investigation determined the patient’s condition to be stable.

## DISCUSSION

In this study, we demonstrated that a patch-based type-II PSG system provided highly concordant sleep and respiratory signals with a traditional PSG in a typical sleep laboratory population. This suggest that the signals recorded by the patch-based PSG had signal characteristics and resolution that were adequate for analysis by an experienced sleep scorer. The CCC was slightly lower on transitional sleep stages N1–N3 compared to other indices of sleep, however, this was expected given the difficulty that scorers have in distinguishing these sleep stages on a traditional PSG.^13^^,^^14^ Nonetheless, N1–N3 still presented moderate (N3) and strong (N1, N2) agreement, while NREM and REM had excellent agreement.

When accounting for the variation in device type and scorer, each scorer’s scoring pattern remained consistent across the patch-based PSG device and traditional PSG (**Table 2**), indicating similar device signal output by the patch-based PSG compared to a traditional PSG. Likewise, the observed CCC and Cohen’s kappa agreement between devices was similar to the observed device ICC and interscorer Cohen’s kappa, indicating that differences in device type are not greatly deviating from interscorer variability rates. We also measured the device set-up time of the patch-based PSG and traditional PSG and the set-up time for the patch-based PSG system was less than 1/3 of that of a traditional PSG.

Compared to a traditional PSG, the patch-based PSG collects EEG from forehead leads only (frontal left and frontal right), and muscle tone is assessed from EMG activity of the masseter rather than submental muscles. Based on previous research, sleep stage scoring derived from forehead EEG leads was comparable to sleep-stage scoring based on in-laboratory traditional PSG recordings and EMG activity derived from the masseter provided additional insights into mandibular movement and muscle activity patterns compared to the submental EMG.^15^^–^^20^ While these studies were done either in normal individuals or utilized small sample sizes, we have extended these findings by providing data from a large, clinical, unselected patient population representative of patients routinely referred for an in-laboratory sleep study. The results of our analysis on a large, clinical population indicate that the differences in device design in respect to the EEG forehead leads and EMG masseter may be negligible as there was good agreement between the patch-based system and traditional PSG system in detecting sleep staging and respiratory events. A deeper investigation into respiratory event detection will follow in the subsequent paper.

The correlation and accuracy in determining sleep stage and AHI, as well as the quick set-up time of the Patch–Based PSG have several important clinical implications. First, they suggest that it is possible to use the patch-based PSG for a routine PSG study. Second, by utilizing a patch-based PSG, the burden placed on the patient during a sleep study can be reduced. Third, the lack of complexity and minimal setup time would enable studies to be performed by patients unattended at home. This would improve access to PSG, especially for patients who do not have easy access to a PSG facility.

Our study design aimed to provide a representative sample of data of sufficient quantity and quality to perform a reliable head-to-head comparison between the patch-based PSG and a traditional PSG. First, to ensure a large, representative sample of ages and sleep disorders within our analysis, we included an unselected population of patients routinely referred for a clinical PSG from 7 sleep centers across Germany. Second, to ensure that our sample had all sleep stages adequately represented, we set a minimum time in bed and a minimum TST for sleep studies and applied an additional strict data quality check. As a result of these strict acceptance criteria, we had a high attrition rate of recruited participants. However, this was an unsurprising finding, as the attrition rates of previous validation studies comparing diagnostic devices have been similar.^21^ We do not believe that the attrition rate has influenced our results as the demographics of the participants that were excluded from the validation study did not differ from those who entered the validation study.

A few limitations are important to mention. First, despite having a good representation of sex and comorbidities within our study group, the population studied was only mildly obese, and most participants recruited were predominantly of a single race. As such, further research will be needed to ensure that patch-based PSG is technically feasible in patients with morbidly obese and in patients from more racially and ethnically diverse populations. Second, the study aimed to validate the patch-based device on a general sleep-disordered population. Thus, further research is required to assess the performance of the patch-based device on different clinical populations. Third, unlike with the traditional PSG, it was not possible to monitor the patch-based PSG throughout the night and make adjustments when leads fell off or signal quality was poor. As such, we had to increase the number of participants recruited to maintain the number of participants that we estimated in our power calculation were necessary for analysis. A real-world study measuring the success rate of the patch-based PSG in the home environment is currently ongoing. Further analyses will be performed to determine if the Onera STS provides sufficient sleep microarchitecture and respiratory event differentiation to lead to a robust diagnosis and treatment plan.

## CONCLUSIONS

In this study, we demonstrated that the patch-based type-II PSG had a similar performance on sleep staging scoring and identification of respiratory events when compared to traditional PSG, thus making it possible to use the patch-based PSG for a routine PSG study. Furthermore, due to its ease of application, the patch-based PSG provides an opportunity for PSG studies to be performed more efficiently across a variety of settings, improving access to PSG to a broader proportion of patients.

## DISCLOSURE STATEMENT

All authors have seen and approved the manuscript. Research and work for this publication was completed by the clinical research organization, Maxis Medical. H. Schneider is a contractor for Onera Health and receives compensation for his time and shareholdings in the company. H. Schneider advised on the design of the clinical trial and played a role in data interpretation. H Schneider did not recruit patients, did not participate in data collection, did not complete data analysis, and did not write the manuscript. All other authors report no conflicts of interest. The study was supported financially by Onera Health, but was independently managed by Maxis Medical Inc, Frankfurt to eliminate company bias, ensure regulatory adherence and adherence with ethical board requirements.

## Supplemental Materials

10.5664/jcsm.11524 Supplemental Materials

## ABBREVIATIONS

AASM: American Academy of Sleep Medicine
AHI: apnea-hypopnea index
CCC: concordance correlation coefficient
EEG: electroencephalography
EMG: electromyography
EOG: electrooculography
ICC: intraclass correlation coefficient
N1: NREM sleep stage 1
N2: NREM sleep stage 2
N3: NREM sleep stage 3
NREM: non-rapid eye movement
Onera STS: Onera Sleep Test System
PSG: polysomnography
REM: rapid eye movement
RMA: reduced major axis
TST: total sleep time

## OPEN ACCESS

Copyright 2025 The Authors. This is an open access article, distributed under the Creative Commons Attribution 4.0 International License. Sharing and adaptation are permitted provided attribution to its original publication in the Journal of Clinical Sleep Medicine is made in accordance with the license.
