# Supplementary material for: Clinical validation of a wireless patch-based polysomnography system
Source: J Clin Sleep Med. 2025 May 1;21(5):813–23. doi: 10.5664/jcsm.11524 (PMC12048320; doi:10.5664/jcsm.11524)
Supplement: Supplemental Materials [file jcsm.11524.sm001.pdf]

Table S1—Clinical Site Recruitment.

**Clinic**	**Count (%)**	**PSG System(s)**
American Sleep Clinic, Frankfurt	56 (16.3%)	Embla S4500 Natus Medical Incorporated
Evang. Kliniken Essen-Mitte	33 (9.6%)	Loewenstein Medical MiniScreen
VAMED Klinik Hagen-Ambrock	71 (20.6 %)	Alice 5 Philips Respironics
Universitätsmedizin Essen Ruhrlandklinik	35 (10.2%)	Nox A1
Lungenzentrum Ulm	49 (14.2%)	Nox A1
Krankenhaus Bethanien	27 (7.8%)	Alice 6 LDe, SONATA Löwenstein Medical
Universitätsklinikum Marburg	73 (21.2%)	Sleep Doc Porti

Site recruitment rates from the 7 participating clinical sites. Included in the table is the PSG system(s) used at each participating site.

Figure S1—CCC and Bland Altman for Wakefulness.

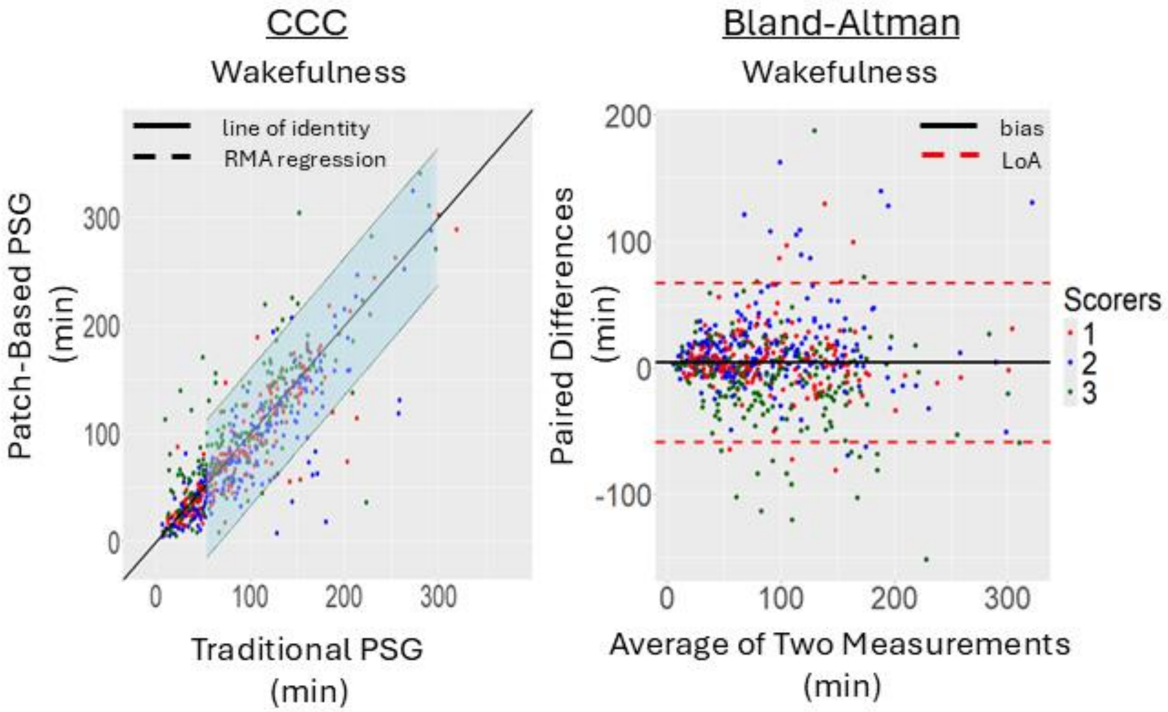

Left: CCC analysis of Wake, in minutes (min), from three scoring centers.
Right: Bland-Altman Plot of Wake time. Figure conventions as described in Figure 3 in the body of the paper.

Figure S2—CCC and Bland Altman for N1.

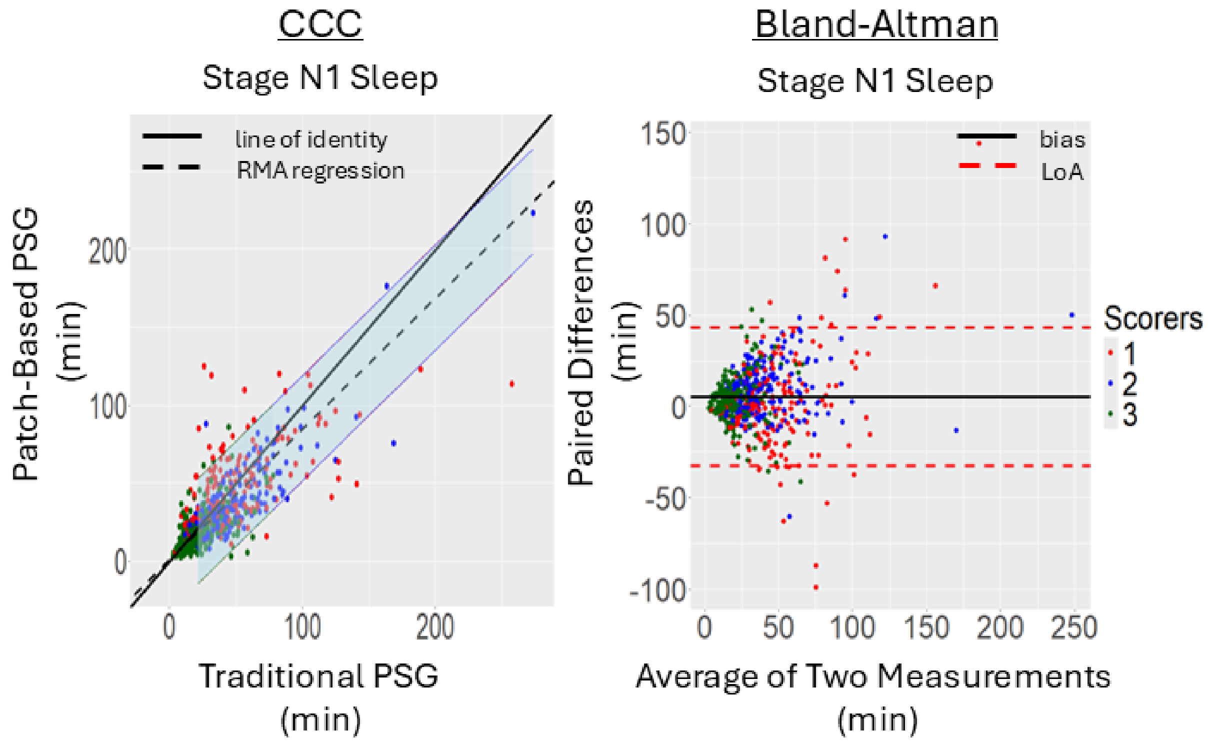

Left: CCC analysis of N1, in minutes (min), from three scoring centers.
Right: Bland-Altman Plot of N1 sleep. Figure conventions as described in Figure 3 in the body of the paper.

Figure S3—CCC and Bland Altman for N2.

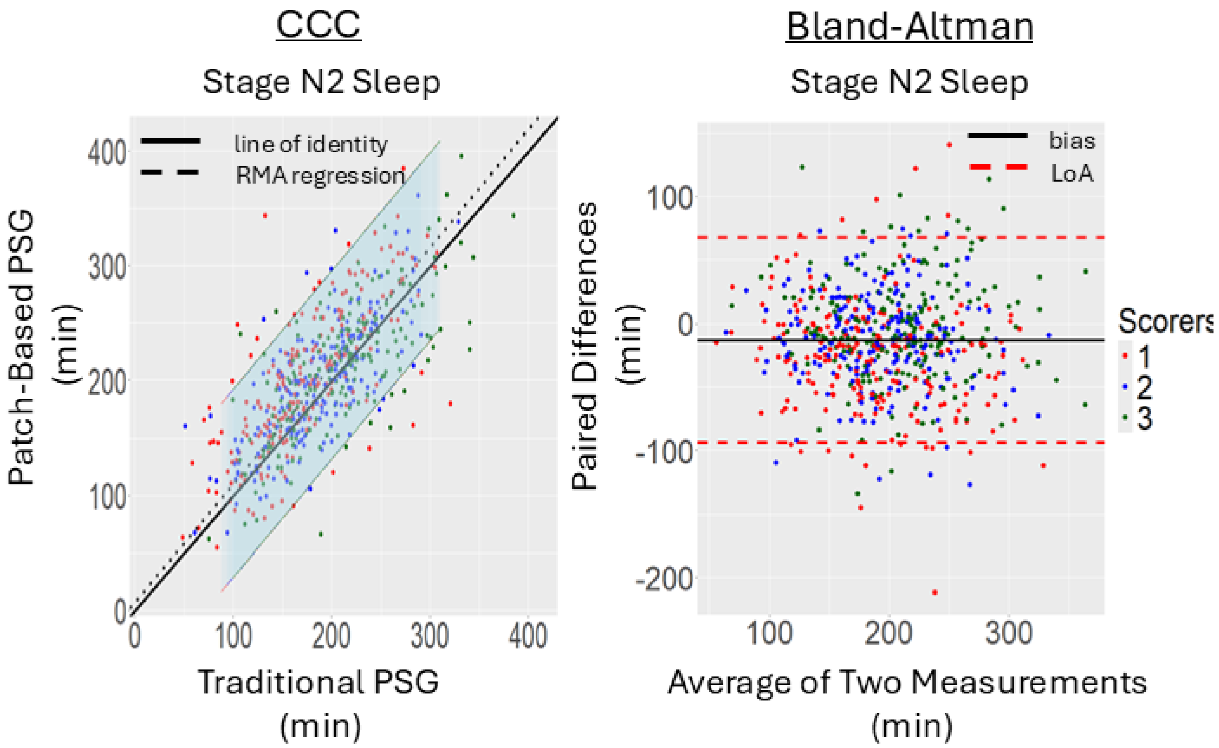

Left: CCC analysis of N2, in minutes (min), from three scoring centers.
Right: Bland-Altman Plot of N2 sleep. Figure conventions as described in Figure 3 in the body of the paper.

Figure S4—CCC and Bland Altman for N3.

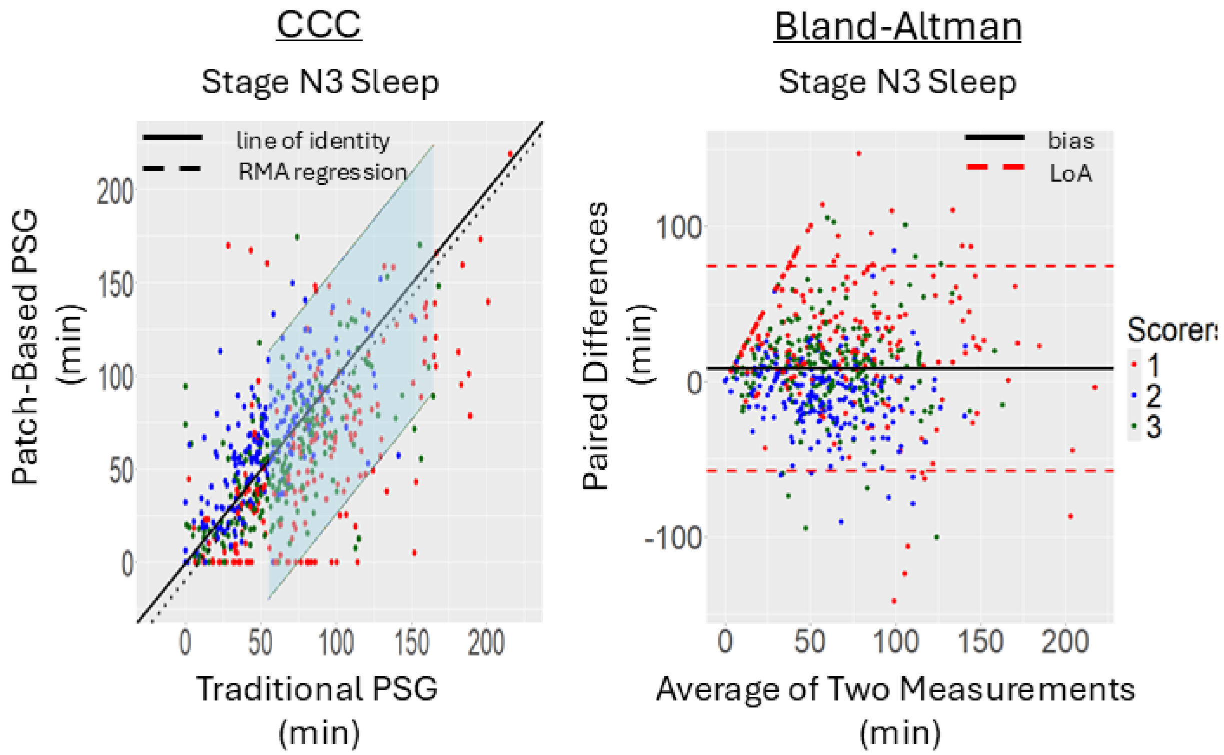

Left: CCC analysis of N3, in minutes (min), from three scoring centers.
Right: Bland-Altman Plot of N3 sleep. Figure conventions as described in Figure 3 in the body of the paper.

Figure S5—CCC and Bland Altman for WASO.

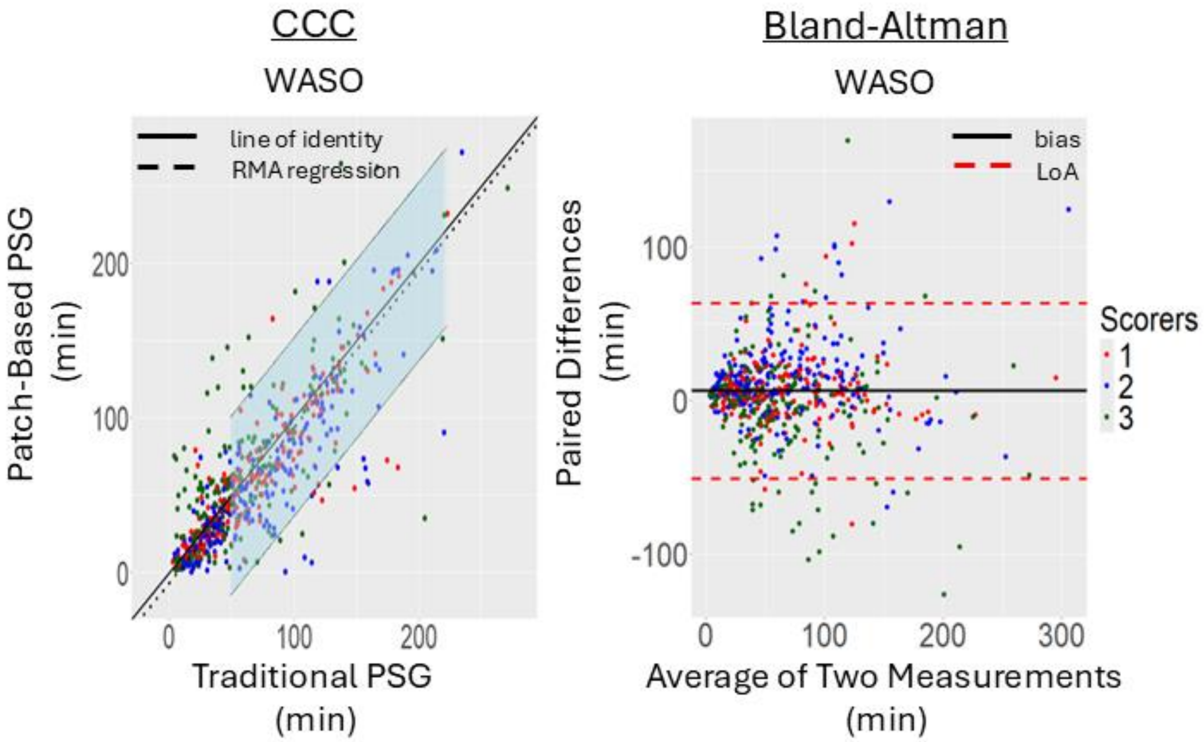

Left: CCC analysis of Wake After Sleep Onset (WASO), in minutes (min), from three scoring centers. **Right**: Bland-Altman Plot of WASO. Figure conventions as described in Figure 3 in the body of the paper.

Figure S6—CCC and Bland Altman for REM Latency.

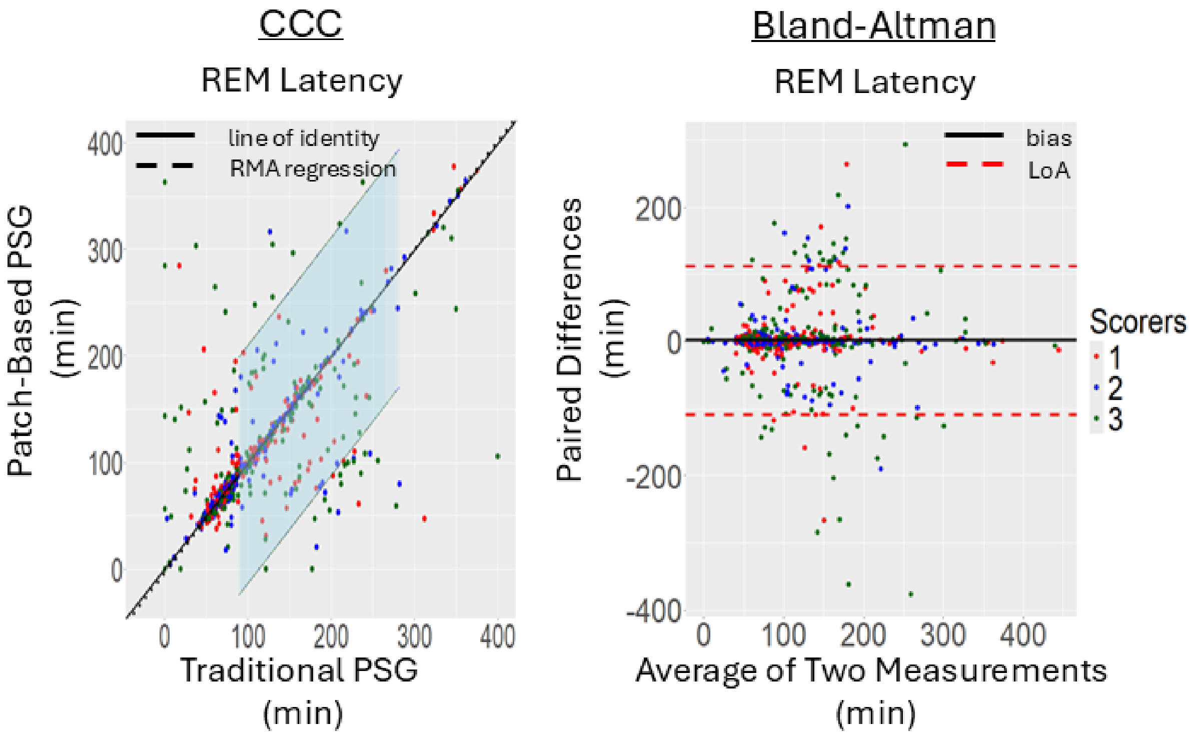

Left: CCC analysis of REM Latency, in minutes (min), from three scoring centers.
Right: Bland-Altman Plot of REM Latency. Figure conventions as described in Figure 3 in the body of the paper.

Figure S7—CCC and Bland Altman for Sleep Onset.

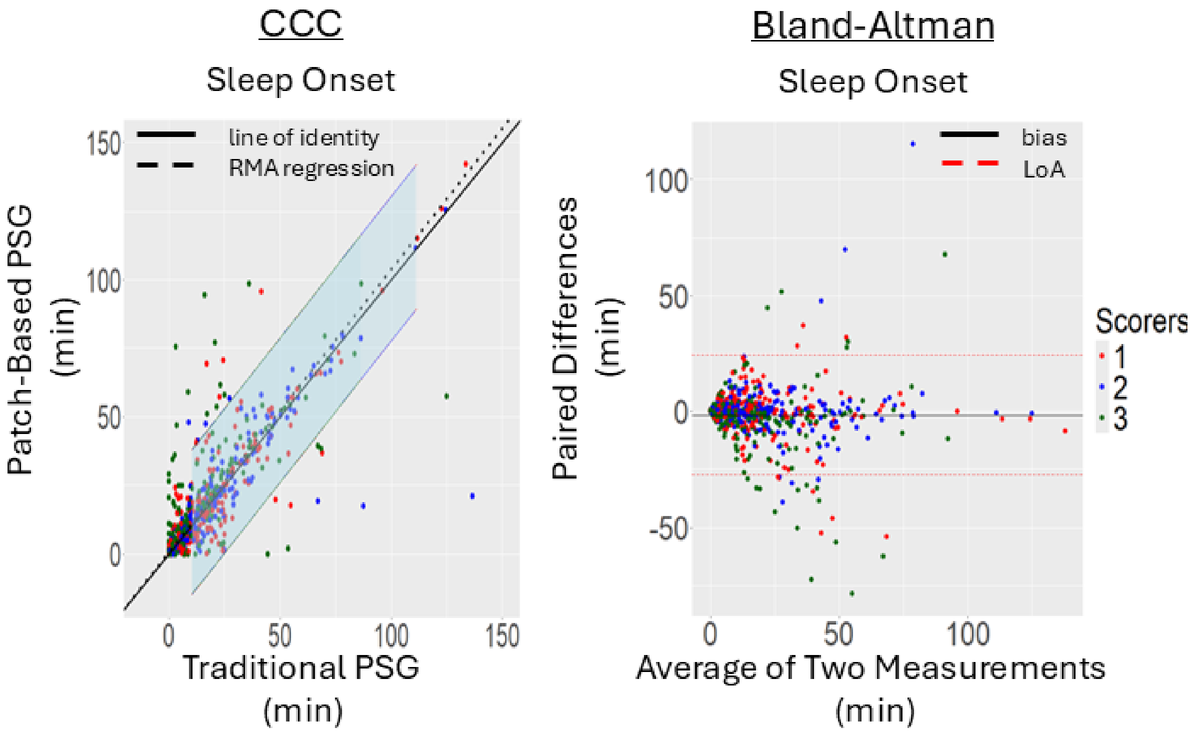

Left: CCC analysis of Sleep Onset, in minutes (min), from three scoring centers.
Right: Bland-Altman Plot of Sleep Onset. Figure conventions as described in Figure 3 in the body of the paper.

Figure S8—CCC and Bland Altman for Sleep Efficiency.

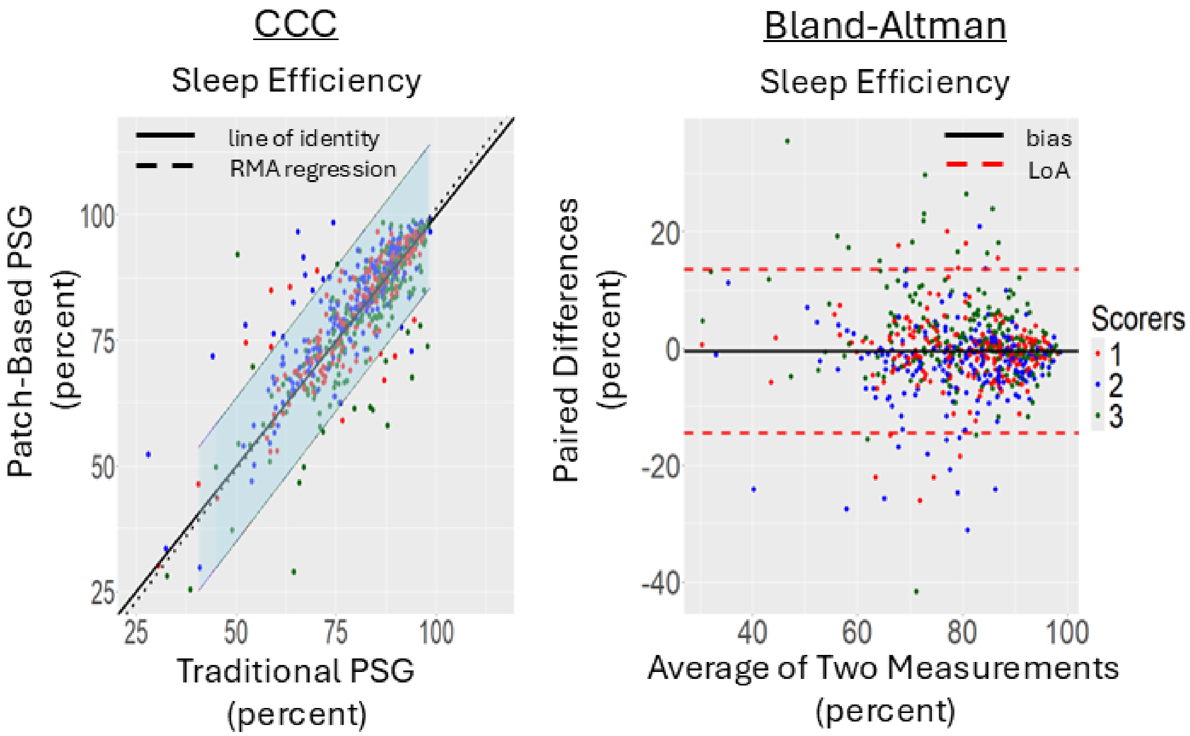

Left: CCC analysis of Sleep Efficiency, as a percent, from three scoring centers.
Right: Bland-Altman Plot of Sleep Efficiency. Figure conventions as described in Figure 3 in the body of the paper.
